# Supplementary material for: Factors Affecting Population Dynamics of Helicoverpa zea (Lepidoptera: Noctuidae) in a Mixed Landscape with Bt Cotton and Peanut
Source: Insects. 2023 Apr 19;14(4):395. doi: 10.3390/insects14040395 (PMC10142863; doi:10.3390/insects14040395)
Supplement: Supplementary file 1 [file insects-14-00395-s001.zip › insects-2220170-supplementary.pdf]

Table S1. Global positioning system (G.P.S.) coordinates for traps on each farm from June 2017 to June 2019. Pheromone trapping was performed in the east (Jackson County) and west (Santa Rosa/Escambia counties) regions of the Florida Panhandle.

Farm code	Region	Crop	Trap 1 (Latitude, Longitude)	Trap 2 (Latitude, Longitude)
BT1	Jackson	Bt cotton	30.871317, -85.018336	30.872386, -85.021106
DR	Jackson	Bt cotton	30.874031, -85.478622	30.873128, -85.476853
DS	Jackson	Bt cotton	30.839078, -85.447517	30.840475, -85.444456
FR1	Jackson	Bt cotton	30.905597, -85.082881	30.904869, -85.082067
FR2	Jackson	Peanut	30.9017, -85.08985	30.904761, -85.089528
PT	Jackson	Peanut	30.865683, -85.145228	30.867381, -85.140558
WB	Jackson	Peanut	30.611589, -84.991003	30.609808, -84.99255
WS	Jackson	Peanut	30.617711, -84.983369	30.617594, -84.981986
BR2	Santa Rosa/ Escambia	Bt cotton	30.907483, -87.432872	30.911625, -87.434503
DL2	Santa Rosa/ Escambia	Bt cotton	30.740631, -87.360261	30.740203, -87.349025
DM	Santa Rosa/ Escambia	Bt cotton	30.933333, -87.170544	30.934764, -87.1602
HE2	Santa Rosa/ Escambia	Bt cotton	30.985889, -87.461592	30.988472, -87.464247
BR1	Santa Rosa/ Escambia	Peanut	30.875667, -87.459228	30.8793, -87.462475
DL1	Santa Rosa/ Escambia	Peanut	30.743639, -87.364242	30.742422, -87.362931
HE1	Santa Rosa/ Escambia	Peanut	30.9816, -87.46605	30.982483, -87.469797
TH	Santa Rosa/ Escambia	Peanut	30.779403, -87.138925	30.778922, -87.135233
